# Effects of a remote mutation from the contact paratope on the structure of CDR-H3 in the anti-HIV neutralizing antibody PG16

**DOI:** 10.1038/s41598-019-56154-y

**Published:** 2019-12-27

**Authors:** Hiroko X. Kondo, Ryo Kiribayashi, Daisuke Kuroda, Jiro Kohda, Akimitsu Kugimiya, Yasuhisa Nakano, Kouhei Tsumoto, Yu Takano

**Affiliations:** 1 School of Regional Innovation and Social Design Engineering, Faculty of Engineering, Kitami Institute of Technology, Kitami, 090-8507 Japan; 2 Department of Biomedical Information Sciences, Graduate School of Information Sciences, Hiroshima City University, Hiroshima, 731-3194 Japan; 3 Laboratory for Computational Molecular Design, RIKEN Center for Biosystems Dynamics Research, Suita, 565-0874 Japan; 4 Medical Device Development and Regulation Research Center, School of Engineering, The University of Tokyo, Tokyo, 113-8656 Japan; 5 Department of Bioengineering, School of Engineering, The University of Tokyo, Tokyo, 113-8656 Japan; 6 Laboratory of Medical Proteomics, Institute of Medical Science, The University of Tokyo, Tokyo, 108-8639 Japan

**Keywords:** Biophysical chemistry, Computational biophysics, Molecular dynamics

## Abstract

PG16 is a broadly neutralizing antibody to the human immunodeficiency virus (HIV). A crystal structure of PG16 revealed that the unusually long 28-residue complementarity determining region (CDR) H3 forms a unique subdomain, referred to as a “hammerhead”, that directly contacts the antigen. The hammerhead apparently governs the function of PG16 while a previous experimental assay showed that the mutation of Tyr^H100Q^ to Ala, which does not directly contact the antigen, decreased the neutralization ability of PG16. However, the molecular mechanism by which a remote mutation from the hammerhead or contact paratope affects the neutralization potency has remained unclear. Here, we performed molecular dynamics simulations of the wild-type and variants (Tyr^H100Q^ to Ala, and Tyr^H100Q^ to Phe) of PG16, to clarify the effects of these mutations on the dynamics of CDR-H3. Our simulations revealed that the structural rigidity of the CDR-H3 in PG16 is attributable to the hydrogen bond interaction between Tyr^H100Q^ and Pro^H99^, as well as the steric support by Tyr^H100Q^. The loss of both interactions increases the intrinsic fluctuations of the CDR-H3 in PG16, leading to a conformational transition of CDR-H3 toward an inactive state.

## Introduction

Antibodies recognize numerous foreign molecules or antigens and are useful in a variety of applications. One area of broad interest in biomedicine is the prevention of infectious disease, with a major focus on the development of vaccines against many viruses^1^. One of the viruses for which we still lack universal vaccines is the human immunodeficiency virus (HIV), which causes acquired immune deficiency syndrome (AIDS); it is already responsible for more than 35 million deaths worldwide^2–4^. Although many researchers are making great efforts^5–8^, the development of HIV vaccines has been hindered by the high mutation rate of HIV genomes, leading to rapid drug resistance^2^. The role of vaccines is to induce antibodies that have desired functions, such as neutralizing activities, and a vaccine is often made from weakened or killed forms of the microbe. To develop an effective vaccine against HIV, the elicitation of broadly neutralizing antibodies against the conserved vulnerable sites on the surface envelope glycoproteins, gp120 and gp41, is required^9^. Toward this ambitious goal, understanding the relationships between the antibody structure, function, and dynamics is essential.

In antibodies, the antigen binding site consists of 6 hypervariable loops, which are also known as complementarity determining regions (CDRs). The specificities of antibodies are governed by the variety of these loop sequences and conformations. Among the 6 CDRs, CDR-H3 is the most diverse in terms of both sequence and structure^10–12^. Accumulating structural information has enabled the classification of 5 of the CDRs (L1, L2, L3, H1, and H2) into the limited number of so-called canonical structures^13–17^; however, the structural classification of the entire CDR-H3 has not yet been feasible^18^. Furthermore, while the lengths of the non-H3 CDRs are limited, those of the CDR-H3 significantly vary, from 3- to 34-residues with an average length of 13.5 for human antibodies^19^ (Kabat/Chothia definition).

**Figure 1:**
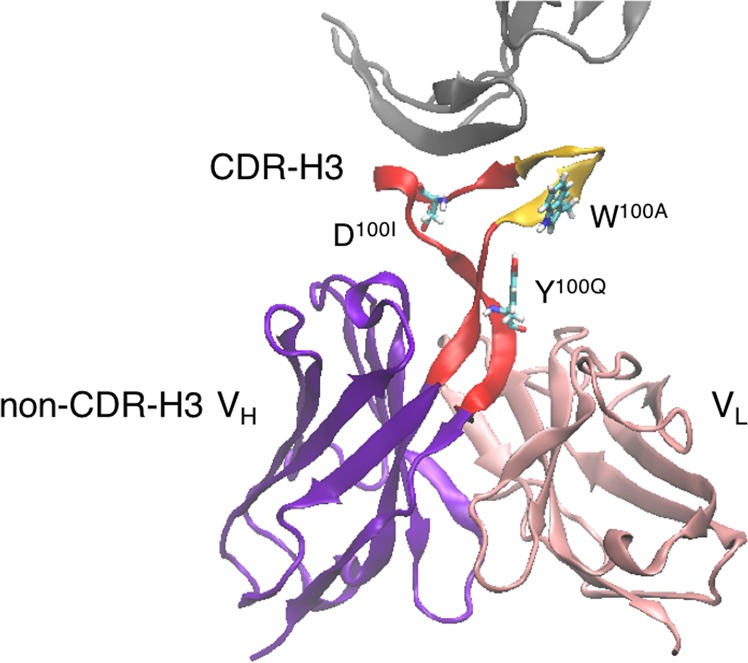
Structure of PG16 used in the simulations (PDB ID: 4DQO). CDR-H3 is colored red and orange (the specificity loop), the other V_H_ domain (non-CDR-H3 V_H_ domain) is purple, and the V_L_ domain is pink. Tyr^H100Q^ is shown as a stick model. To highlight the relationship between the position of Tyr^H100Q^ and the antigen, the antigen molecule is represented as a gray cartoon model, but it was not included in the simulations.

Numerous reports have delineated the structural basis of the neutralizing activities of antibodies against HIV^20^. Among the neutralizing antibodies, PG16 was discovered to have the ability to neutralize ~80% of HIV isolates, by binding to novel epitopes preferentially expressed on the surface envelope glycoprotein^21^. Recently, the crystal structure of PG16 was determined at 2.4 Å resolution, and revealed that PG16 possesses an unexpectedly long CDR-H3 consisting of 28 residues (Kabat/Chothia definition)^22^ (Fig. 1). The CDR-H3 protrudes from the variable domain, and this protruded portion was referred to as a “hammerhead”. The C-terminal region of the CDR-H3 has a typical kinked conformation^15,23^ and is supported by several contacts between the variable domains of the light and heavy chains (light variable domain (V_L_ domain) and the non-CDR-H3 heavy variable domain (non-CDR-H3 V_H_ domain)), as shown in Fig. 1.

Despite the structural diversity of the CDR-H3s in antibodies in general^10^, based on the crystal structures of the antigen-bound and unbound states^22,24^, PG16 did not undergo a large conformational change upon antigen binding. The hammerhead structure of the CDR-H3 is thought to be stable, due to the 13 hydrogen bonds within the H3. Experimental mutagenesis studies suggested that a 7-residue “specificity loop” within the hammerhead subdomain of the CDR-H3, consisting of Ile^H100^, Trp^H100A^, His^H100B^, Asp^H100C^, Asp^H100D^, Val^H100E^, and Lys^H100F^, plays a significant role in the specificity and neutralization potency^22^. In addition, PG16 was surprisingly tolerant of mutations within the CDRs, and was compromised only by the mutations of Trp^H100A^, Asp^H100I^, and Tyr^H100Q^ in the CDR-H3^22^. The fold increases in the IC_50_ for the PG16 variants containing the single Ala substitution of Trp^H100A^, Asp^H100I^, and Tyr^H100Q^ were >50, 200, and 44, respectively, relative to the wild-type^22^. These experimental observations indicated that the three residues are directly involved in the neutralization activity. The deleterious effects could be explained partially by the positions of the mutated residues: Trp^H100A^ and Asp^H100I^ are located on or in the vicinity of the specificity loop and are spatially close to the antigen (Fig. 1). However, while there is a single hydrogen bond between Tyr^H100Q^ and Pro^H99^, Tyr^H100Q^ is farther apart from the specificity loop and contact paratopes (Fig. 1), and hence its physical role remains unclear.

Recent advancement in computational powers and algorithms have made it possible to use computer simulations to interpret experimental results and further generate testable hypotheses^25,26^. Molecular dynamics (MD) simulations can reveal the effects of amino acid mutations on a protein structure^27–29^. Schmidt *et al*. examined correlations between somatic mutations of neutralizing antibodies against influenza virus and their affinities for the antigens on the basis of computational and experimental studies, and proposed a possible mechanism of affinity maturation^30^. Ovchinnikov *et al*. used MD simulations to study the effect of somatic mutations observed in three different neutralizing antibodies against HIV, and showed that mutations at the framework regions could rigidify the antibodies as affinity maturation progresses^31^. These analyses indicate that MD simulations can reveal dynamic influences that cannot be detected by analysis of a static crystal structure and can be used to predict the functional effects of mutations on protein structures.

In this study, we investigated the effects of mutations of Tyr^H100Q^ on the structure and dynamics of PG16, by MD simulations. To directly clarify the effect of a mutation diminishing the neutralizing activity, we performed the three independent 300-ns simulations of the PG16 F_V_ fragment (Fig. 1) of the inactive mutant of Tyr^H100Q^ to Ala (Y100qA), as well as the wild-type (WT). In addition, to solely assess the importance of the hydrogen bond between Tyr^H100Q^ and Pro^H99^, we also included the mutant of Tyr^H100Q^ to Phe (Y100qF) in our simulations. Our simulations revealed the increased fluctuation of CDR-H3 in both mutant systems, suggesting that the loss of the hydrogen bond between Tyr^H100Q^ and Pro^H99^ in the mutant systems could enhance the inherent fluctuation of CDR-H3. Especially, the large conformational change of the CDR-H3 and the formation of other hydrogen bonds between CDR-H3 and the other region in the heavy chain were observed in 2 out of the 3 simulations of the Y100qA system. These results imply that the mutation of Tyr^H100Q^ to Ala prevents PG16 from recognizing HIV by the conformational transition of the CDR-H3 toward an inactive state, resulting in the reduction of the neutralization potency.

## Results

### Stability of the systems during the simulations

**Figure 2:**
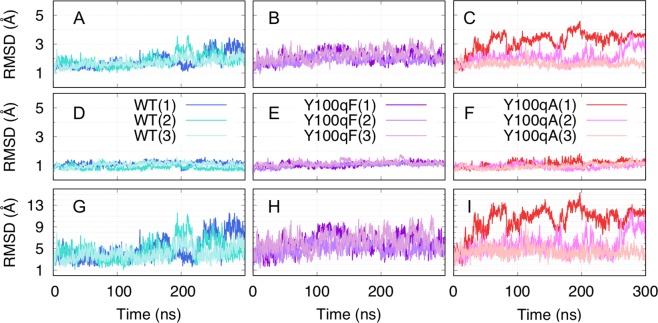
Time sequences of the RMSDs of the main-chain atoms from the crystal structure in (**A**–**C**) the whole F_V_ domain, (**D**–**F**) the non-CDR-H3 V_H_ domain, and (**G**–**I**) the CDR-H3. The blue, turquoise, and light-turquoise represent the WT trajectories, dark-violet, purple, and plum represent Y100qF trajectories, and red, orchid, and pink represent Y100qA trajectories, respectively. In (**A**–**C**), the RMSDs were calculated after fitting the main-chain atoms of the whole F_V_ domain to the reference crystal structure. In (**D**–**F**) and (**G**–**I**), the RMSDs were calculated after the least squares fitting for the main-chain atoms of the non-CDR-H3 V_H_ domain.

To confirm the stability of the systems during the simulations, we calculated the root mean square deviation (RMSD) from the crystal structure. The time courses of the RMSD values in the trajectories of the WT and two mutant systems were plotted every 200 ps, as illustrated in Fig. 2. We calculated the RMSDs for the main-chain atoms (N, Cα, C, and O) in the entire protein by fitting those atoms to the crystal structure by rotation and translation (least squares fitting) (Fig. 2A–C). The mean RMSD values for the whole F_V_ domain were 1.84 ± 0.51 Å, 1.84 ± 0.45 Å, and 1.73 ± 0.30 Å for the WT, 2.13 ± 0.41 Å, 1.89 ± 0.29 Å, and 2.23 ± 0.42 Å for the Y100qF mutant, and 3.18 ± 0.64 Å, 2.09 ± 0.46 Å, and 1.63 ± 0.22 Å for the Y100qA mutant, respectively, suggesting that the protein structures became slightly more flexible in the mutant systems. To examine which part becomes more flexible in the mutant systems, the RMSDs of two regions, the main-chain atoms of the CDR-H3 (red and orange in Fig. 1) and those of the non-CDR-H3 V_H_ domain (purple in Fig. 1) were calculated, after the least squares fitting for the main-chain atoms of the latter. As shown in Fig. 2D–F, the non-CDR-H3 V_H_ domain was very stable in all of the systems, as the mean RMSD values were 1.13, 0.85, and 1.07 Å for the WT, 1.07 Å, 1.03 Å, and 1.16 Å for the Y100qF, and 1.11 Å, 0.95 Å, and 1.05 Å for the Y100qA. In contrast, the RMSD values of the CDR-H3 in the mutant systems tended to be larger than those in the WT (Fig. 2G–I), suggesting greater fluctuations of the CDR-H3 upon mutation, as the mean values were 4.86 ± 2.15 Å, 4.71 ± 1.88 Å, and 4.06 ± 1.24 Å for the WT, 6.10 ± 1.75 Å, 5.06 ± 1.22 Å, and 6.39 ± 1.61 Å for the Y100qF, and 10.28 ± 2.35 Å, 5.71 ± 1.83 Å, and 4.43 ± 0.99 Å for the Y100qA systems, respectively. These results also suggested that the CDR-H3 of the Y100qF mutant is more rigid than that of the Y100qA mutant, probably because the presence of the phenyl group generates steric support for the hammerhead subdomain, and that Tyr^H100Q^ is important for the conformational maintenance of the CDR-H3.

### Differences in the fluctuation among the three systems

**Figure 3:**
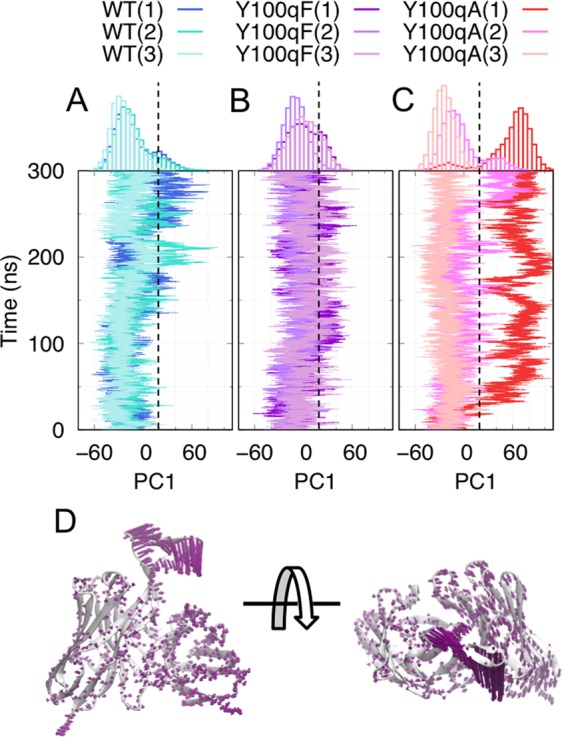
The PCA results and dominant motions in the simulations. (**A**–**C**) Time sequences of the PC1 values of the WT (**A**), Y100qF (**B**), and Y100qA (**C**) systems. The histograms on the top of the plots show the number of snapshots in each interval along PC1 for each trajectory. The black dashed lines represent that PC1 value equals 20. The coloring is the same as in Fig. 2. (**D**) The first eigenvector is drawn as arrows onto each atom in the average structure (side view, left; top view, right).

In order to analyze the details of the structural fluctuations of the mutant proteins, we performed a principal component analysis (PCA)^32,33^ for the Cartesian coordinates of the proteins in a combined trajectory (9 simulations in total) of the three systems (WT, Y100qF, and Y100qA). The projections of each trajectory onto the first eigenvector (PC1), whose contribution was 32.87%, are plotted along with time in Fig. 3A–C; the larger PC1 values tended to be distributed in the two mutant systems, indicating that PC1 represented a characteristic difference between the WT and two mutant systems. In the Y100qA simulations, two of the three trajectories showed bimodal distribution with a valley around the PC1 value of 20, suggesting a conformational transition. In contrast, in the Y100qF simulations, two of the three trajectories had wide bell-shaped distributions, and, in the WT simulations, two of the three trajectories had heavy-tailed distributions, suggesting that the structure fluctuated around the native state in the WT simulations, and, compared to the WT, the fluctuation became larger in the Y100qF systems. The PC1 represented the twisting and bending motions of the CDR-H3 toward the V_H_ and V_L_ domains (Fig. 3D), and the PC2 showed the sliding motions of the V_L_ and CDR-H3 domain (Fig. S1).

Since the large motions in each system might have been lost as a result of merging the trajectories, we also conducted the PCA of the trajectories for each system (WT, Y100qF, and Y100qA), and compared these results with the PC1 from the combined trajectory (PC1_combined). The degrees of similarity between the PC1_combined and the PC1 obtained from each trajectory were evaluated by the inner product of two vectors, and were 0.91, 0.99, and 0.82 for the WT, Y100qF, and Y100qA systems, respectively. The similarity between the PC1s of the two mutant systems was 0.75.

Together, these results suggest that the effects of the two mutations (Y100qF and Y100qA) on the structural fluctuations were similar, and the direction of the structural changes observed in these two mutant systems somewhat corresponds to the dominant fluctuation mode observed in the WT. To obtain a finer view of the roles of these mutations, we analyzed the trajectories at atomic resolution, as shown below.

### Structural difference between the WT(3) and mutant systems

**Figure 4:**
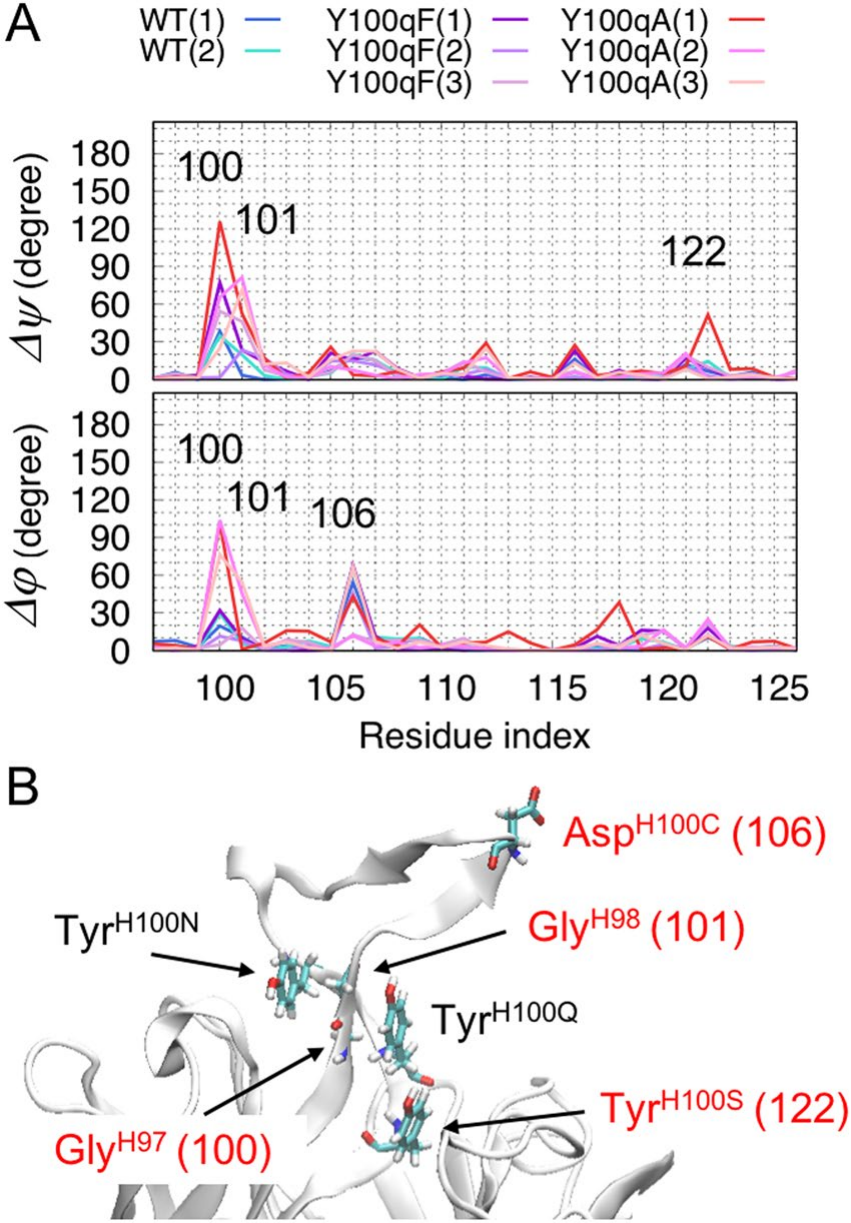
(**A**) Distributions of differences in dihedral angles, Δ*ψ* (top panel) and Δ*φ* (bottom panel), of residues from the 97^th^ to 126^th^ indices. The results of WT are shown in blue and turquoise, Y100qF in dark-violet, purple and plum, and Y100qA in red, orchid, and pink. (**B**) The locations and the corresponding indices of the residues showing large differences in the dihedral angles (Δ*ψ*, Δ*φ* > 50°) from the WT(3) are represented as stick models and labeled with red letters (Gly^H97^, Gly^H98^, Asp^H100C^, and Tyr^H100S^). The residues composing the aromatic core are also shown as stick models and labeled with black (Tyr^H100N^, and Tyr^H100Q^) and red (Gly^H97^) letters.

In order to clarify the structural difference between the WT and mutant systems, we first analyzed the dihedral angles (*φ*, *ψ*) and calculated the difference between the WT(3) and the other systems. We chose the WT(3) as a reference because the protein structure during the simulations were most stable among the three WT simulations, and the distribution of the dihedral angles would represent dynamical characters of the protein. Many of residues showing large differences in the dihedral angles from the WT(3) were around CDR-H3 (Fig. S2), and they are shown in Fig. 4A, in which the residue indices are represented by serial numbers starting from 1, and those of CDR-H3 are from 98 to 125. For example, Tyr120 represents Tyr^H100Q^. The dihedral angles at the mutated residue (index of 120) of all the systems have almost the same values as those of the WT(3), indicating that the conformational change did not occur at the mutated residue itself. In Fig. 4B, the locations and the residue numbers of the residues showing large differences in the dihedral angles from the WT (Δ*ψ*, Δ*φ* > 50°) are represented as stick models and labeled with red letters. As shown in Fig. 4A, the 100^th^ residue, which corresponds to Gly^H97^, had the largest values for both Δ*ψ* and Δ*φ*. For Δ*ψ* of the 100^th^ and/or 101^st^ residues (Gly^H97^ and Gly^H98^, respectively), the trajectories of the mutant systems showed larger values as compared to those of the WT: the sums of Δ*ψ* of 100^th^ and 101^st^ residues averaged over three trajectories in each system were 48.37 ± 6.37° for WT, 75.61 ± 35.53° for Y100qF, 140.65 ± 33.62° for Y100qA. On the other hand, for Δ*φ* of the same residues, the trajectories of Y100qA(1-2) showed larger values whereas Y100qA(3) showed a lower value as compared to the trajectories of WT(1-2) and Y100qF(1-3) most likely because conformational transitions were not observed during the simulation of Y100qA(3). These results indicate that the changes in the dihedral angles of Gly^H97^ and Gly^H98^ lead to twist and bending motions of CDR-H3 in the mutant systems. As evidenced by the crystal structures^22^, Gly^H97^ and Gly^H98^ are stabilized by aromatic interactions with Tyr^H100N^ and Tyr^H100Q^ (Fig. 4B), and hereafter we refer to these residues as the “aromatic core”. The mutation of Tyr^H100Q^ may have loosened the aromatic core and caused the fluctuation of Gly^H97^.

In a previous study, Pejchal *et al*. showed that, in addition to the increases by the mutations of Trp^H100A^, Asp^H100I^, and Tyr^H100Q^, a slight increase in the IC_50_ relative to the WT was observed in the mutation of Tyr^100N^ to Ala, whereas there was no such change in the mutation of Tyr^100N^ to Phe^22^. These results suggested that the interaction between the two aromatic residues (Tyr^H100N^/Phe^H100N^ and Tyr^H100Q^) is one of the important factors in the neutralizing potency. However, considering the slight increase in the IC_50_ (~11 fold) of the mutation of Tyr^100N^ to Ala, it is still unclear why the mutation of Tyr^100Q^ to Ala led to the significant increase in the IC_50_ (~44 fold). In the crystal structures, a prominent difference between Tyr^H100Q^ and Tyr^H100N^ is the presence or absence of a hydrogen bond; there is a hydrogen bond between the side-chain of Tyr^H100Q^ and the backbone of Pro^H99^, whereas no such interactions between Tyr^H100N^ and the surrounding residues exist. To clarify the specific role of the hydrogen bond, we examined the frequencies of hydrogen bond formation during the simulations in each system, as shown below.

### Effects of a mutation on hydrogen bond formation in CDR-H3

We analyzed the differences in the hydrogen bond formation around the CDR-H3 between the WT(3) and the other trajectories. The hydrogen bonds in each trajectory were identified by the wernet_nilsson method in the MDTraj library^34^. Hydrogen bond formation in a residue pair in each trajectory was defined as the ratio of the number of snapshots in which a hydrogen bond is formed in the residue pair to the number of all snapshots. The differences in hydrogen bond formation between the WT(3) and another system (*k*) were defined as the ratio^*k*^ minus the ratio^WT(3)^.

**Figure 5:**
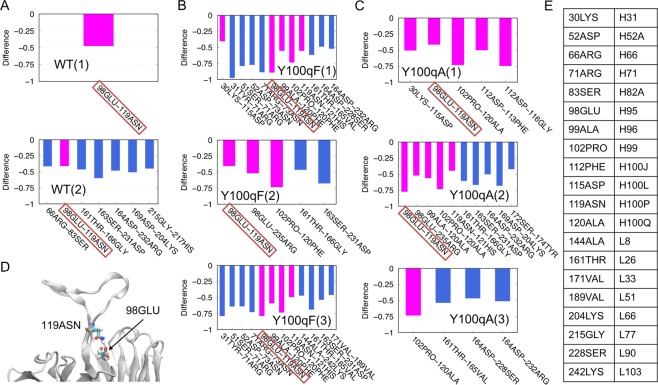
(**A–C**) The differences in the ratios of hydrogen bond formation of (**A**) WT(1-2), (**B**) Y100qF(1-3), and (**C**) Y100qA(1-3) from the WT(3). Only the “decreased pairs” (difference in ratio <−0.4) are plotted. The pairs including one or more residues in the CDR-H3 are colored magenta. The pair included in most of the trajectories (7 out of 8) is enclosed in red boxes. (**D**) The locations of 98GLU and 119ASN are represented as stick models. (**E**) Correspondence table of the residue indices used in A-C (left) and the Kabat numbering (right).

The “decreased pairs” are shown in Fig. 5, in which the differences in the ratio between each trajectory and the WT(3) are less than −0.4. The pairs, including residues in CDR-H3, are shown in magenta. These figures revealed that many of the “decreased” hydrogen bond formation occurs in the CDR-H3. In the Y100qA(1, 2), the loss of the hydrogen bonds involved in the CDR-H3 led to its deformation. The decreased residue pair observed in most of the trajectories (7 out of 8) was 98GLU-119ASN (Fig. 5A–C). Considering the location in the structure (Fig. 5D), this hydrogen bond may also play a role in stabilizing the conformation of CDR-H3. As expected, the largest decrease in hydrogen bond formation was observed between the Pro102 (Pro^H99^) and Ala/Phe120 (Ala^H100Q^/Phe^H100Q^) pair in both mutants. The loss of the hydrogen bond would have a destabilizing effect even on Phe^H100Q^, in spite of the steric support by the aromatic side-chain. However, the root mean square fluctuation (RMSF) of the side-chain heavy atoms of Phe^H100Q^ in the Y100qF mutant was smaller than that of the WT (Tyr^H100Q^): 1.28 ± 0.02 Å for the Y100qF trajectories and 1.49 ± 0.29 Å for the WT trajectories. In contrast, the fluctuation of Tyr^H100N^ (the other component of the aromatic core) became larger in the mutant systems, as the RMSF values of the side-chain of Tyr^H100N^ were 1.60 ± 0.44 Å, 1.92 ± 0.79 Å, and 1.86 ± 0.48 Å for the WT, Y100qF, and Y100qA systems, respectively. The RMSF values of side-chain heavy atoms of each residue are shown in Fig. S3.

These results suggest that the role of the aromatic residues is to maintain the stability of the aromatic core. However, as seen in the case of the Y100qF mutant, the loss of the hydrogen bond between Tyr^H100Q^ and Pro^H99^ could lead to the larger fluctuation of Tyr^H100N^. When the steric support by the aromatic ring disappears by the Ala mutation, the fluctuation of Tyr^H100N^ becomes more evident. As a consequence, the destabilized aromatic core would result in the instability of CDR-H3.

We also analyzed the “increased pairs”, in which the difference in the ratio of hydrogen bond formation from the WT(3) is >0.4 for both mutant systems and the other WT systems (Fig. S4). In the trajectory of Y100qA(1), Tys111 (sulfonated tyrosine) tended to form hydrogen bonds with Tyr31. This interaction was formed in the simulated structure with twisting and bending motions of CDR-H3, which corresponded to conformational changes along the PC1 (Fig. 3D) and was not observed in the simulations of the WT and the Y100qF mutant.

## Discussion

The CDR-H3s in antibodies play a pivotal role in antigen recognition. Previous studies have suggested the correlations between the stability of the CDR-H3 in antibodies and the neutralizing potency for the antigenic site of HIV strains^30,35,36^. To generate higher affinity antibodies, artificial mutations are often designed at antibody-antigen interfaces^37^. Beneficial mutations could be identified at an antibody-antigen interface, based on the complex crystal structure. However, stabilizing CDR-H3 alone may be another strategy to enhance the binding affinities for target antigens, especially when the CDR-H3 is long, as seen in the anti-HIV neutralizing antibody PG16.

In the present study, to explore the relationship between a deleterious mutation (Y100qA) and the dynamics of CDR-H3, we performed MD simulations on the F_V_ region of PG16 in three systems: the WT, and the Y100qF and Y100qA mutants. As a result, a conformational transition (bending motion of CDR-H3) was observed in 2 out of the 3 simulations of the Y100qA mutant system. Similarly, in the Y100qF systems, fluctuations along the same reaction coordinate were observed to various extents, and, compared to the WT, the loss of the single hydrogen bond by the Phe mutation at the H100Q position conferred more flexibility to CDR-H3. However, the presence of a phenyl group sterically supported the CDR-H3, and therefore it was more rigid than that in the corresponding Ala mutant. The Ala mutation simultaneously lost the steric support and the hydrogen bond, resulting in the large deformation of the CDR-H3 in the Y100qA mutant. Overall, in both mutant systems, the dihedral angles of the main-chain (*φ*, *ψ*) of Gly^H97^ largely changed, as compared to those in the WT (Fig. 4A), and this change would be involved in the bending motion of CDR-H3. Analysis of hydrogen bond formations also suggests that the hydrogen bond between Glu^H95^ (98GLU) and Asn^H100P^ (119ASN) may play a role in the stabilization of the CDR-H3 structure.

Interestingly, the direction of the bending motions observed in the mutant systems corresponded to the direction of the intrinsic motions observed in the WT. This is reminiscent of the population shift mechanism of molecular recognition and protein allostery. The population shift is a view of the relationships between the functional motions and the intrinsic fluctuations of unbound-state proteins^38^. A variety of functional motions, such as ligand binding, have been interpreted by population shift mechanisms^39,40^. In this context, our results showed that the dominant intrinsic fluctuation of the unbound-state PG16 was enhanced, even by single point mutations (Y100qA and Y100qF) (Fig. 3A–C). A previous experimental assay demonstrated that the mutation of Y100qA diminished the neutralizing potency^22^, implying the delicate balance between the intrinsic functions of PG16 and the functional dynamics.

In addition to the structural rearrangement of CDR-H3, the analysis of hydrogen bond formation also suggested the possibility that the hydrogen bonds between Tys111 and Tyr31, which were not observed in the WT simulation, also prevent antigen recognition by the Y100qA mutant. These results could explain the experimental result that the neutralizing ability was significantly decreased by the Y100qA mutation (the fold increase in IC_50_ relative to WT was 44), although it is far from the antigen binding site or contact paratope^22^.

## Methods

### System setup

In this study, we considered three systems: the wild-type system (WT), the Tyr^H100Q^ to Ala mutated system (Y100qA), and the Tyr^H100Q^ to Phe mutated system (Y100qF). The initial structures of the simulations were prepared by using the atomic coordinates of the PG16 antibody determined by X-ray crystallographic studies: the PG16 Fab fragment complexed with the V1/V2 region from the HIV strain ZM109 at 2.44 Å resolution (PDB entry: 4DQO)^24^. Only the F_v_ regions (1^st^ to 137^th^ residues of chain H and 1^st^ to 111^st^ residues of chain L) of the Fab fragments (Fig. 1) were used, and all crystal water molecules and sugar units were removed. All ionizable side chains were configured in their characteristic ionized states at pH 7.0. The Cys pairs that form a disulfide bond were connected to each other. Each system was solvated in a rectangular box with dimensions of approximately 70 × 85 × 80 Å^3^. Three sodium ions were added to neutralize the system. All systems were composed of approximately 45,000 atoms.

### Simulation details

All simulations were conducted with the GROMACS software, version 4.6.4 and 4.6.7^41^. The periodic boundary condition was applied, and electrostatic interactions were treated by using the Particle Mesh Ewald (PME) method^42^ with a grid spacing of 0.16 nm. A cutoff distance of 1.0 nm was used for the Ewald real space and van der Waals truncation. The non-bonded interactions were updated every step for the minimization and every 10 steps for the MD simulations. The Amber ff99SB-ILDN force field^43^ was applied for normal amino acids and sodium ions, the generalized Amber force field (GAFF)^44^ was used for the sulfonated Tyr, Tys, and the extended simple point charge (SPC/E) model^45^ was applied for the water molecules. The simulation protocols were the same for all systems.

We first performed the energy minimizations on the whole system, and gradually heated the system to 300 K during 200 ps (30 K/20 ps) using the V-rescale thermostat^46^. Subsequently, two equilibration processes were applied: 100-ps simulations with the position restraints of the heavy atoms to relax the hydrogen atoms and with the position restraints of the backbone atoms of the whole F_V_ domain to relax the side-chain conformations. The force constant was 1,000 kJ mol^−1^ nm^−2^. We performed each 300-ns production run under NPT (constant pressure and constant temperature) conditions, with a time step of 2 fs. In each MD simulation, temperature and pressure were maintained at 300 K and 1 bar with the Nosé–Hoover thermostat^47–49^ and the Parrinello–Rahman barostat^50,51^, respectively. All bonds were constrained by the LINCS algorithm^52^. Three simulations with different initial velocities were performed for each system.

### Trajectory analysis

We defined each domain as follows: non-CDR-H3 V_H_ domain: 1^st^ to 97^th^ and 126^th^ to 137^th^ residues (purple in Fig. 1), CDR-H3: 98^th^ to 125^th^ residues (shown in red in Fig. 1), and V_L_ domain: 138^th^ to 248^th^ residues (pink in Fig. 1). Molecular figures were produced with the visual molecular dynamics (VMD) software^53^. The RMSF values were calculated for side-chain heavy atom after the least squares fitting for the Cα atoms of the F_V_ domain and averaged over the atoms in each residue. For PCA, the snapshots were sampled every 20 ps during the 300-ns simulations, and 15,001 snapshots were obtained for each trajectory. The snapshots from all the trajectories were combined and superposed to the average structure by using MDTraj library^34^. The coordinates of the main-chain atoms of the non-CDR-H3 V_H_ domain were used for the least squares fitting. After removal of the rotational and translational motions, a positional covariance matrix of main-chain atoms of the whole F_V_ domain was calculated. The PCA was carried out by using scikit-learn Python library.

## Supplementary information

Supplementary Information
