# Supplementary material for: Effects of a remote mutation from the contact paratope on the structure of CDR-H3 in the anti-HIV neutralizing antibody PG16
Source: Sci Rep. 2019 Dec 27;9:19840. doi: 10.1038/s41598-019-56154-y (PMC6934664; doi:10.1038/s41598-019-56154-y)
Supplement: Supplementary file 1 — Supplementary Information [file 41598_2019_56154_MOESM1_ESM.docx]

**Effects of a remote mutation from the contact paratope on the structure of CDR-H3 in the anti-HIV neutralizing antibody PG16**

Hiroko X. Kondo^1,2,3,*^, Ryo Kiribayashi^2^, Daisuke Kuroda^4,5,*^, Jiro Kohda^2^, Akimitsu Kugimiya^2^, Yasuhisa Nakano^2^, Kouhei Tsumoto^4,5,6^ & Yu Takano^2,*^

^1^ School of Regional Innovation and Social Design Engineering, Faculty of Engineering, Kitami Institute of Technology, 165 Koen-cho, Kitami, 090-8507, Japan

^2^ Department of Biomedical Information Sciences, Graduate School of Information Sciences, Hiroshima City University, Hiroshima 731-3194, Japan

^3^ Laboratory for Computational Molecular Design, RIKEN Center for Biosystems Dynamics Research, 6-2-3, Furuedai, Suita, 565-0874, Japan

^4^ Medical Device Development and Regulation Research Center, School of Engineering, The University of Tokyo, Tokyo 113-8656, Japan

^5^ Department of Bioengineering, School of Engineering, The University of Tokyo, Tokyo 113-8656, Japan

^6^ Laboratory of Medical Proteomics, Institute of Medical Science, The University of Tokyo, Tokyo 108-8639, Japan

**Correspondence details:**

Yu Takano, Ph.D.

Graduate School of Information Sciences, Hiroshima City University, Hiroshima 731-3194, Japan

Phone: +81-82-830-1825

[ytakano@hiroshima-cu.ac.jp](mailto:ytakano@hiroshima-cu.ac.jp)

Hiroko X. Kondo, Ph.D.

Faculty of Engineering, Kitami Institute of Technology, Kitami, 090-8507, Japan

Phone: +81-157-26-9401

[h_kondo@mail.kitami-it.ac.jp](mailto:h_kondo@mail.kitami-it.ac.jp)

Daisuke Kuroda, Ph.D.

Medical Device Development and Regulation Research Center, School of Engineering, The University of Tokyo, Tokyo 113-8656, Japan

Phone: +81-3-6409-2129

[d-kuroda@protein.t.u-tokyo.ac.jp](mailto:d-kuroda@protein.t.u-tokyo.ac.jp)


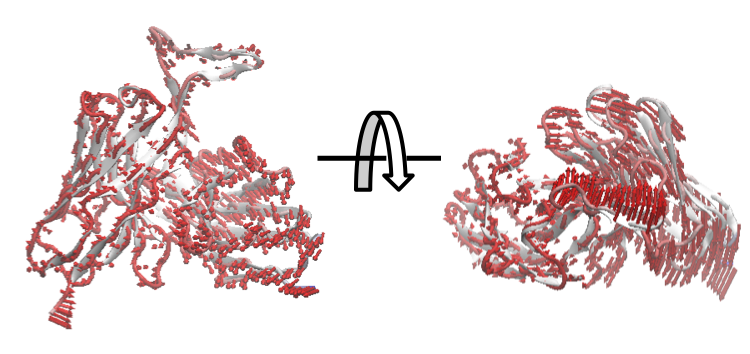


**Figure S1.** The second eigenvector is drawn as arrows onto each atom in the average structure (side view: left and top view: right)


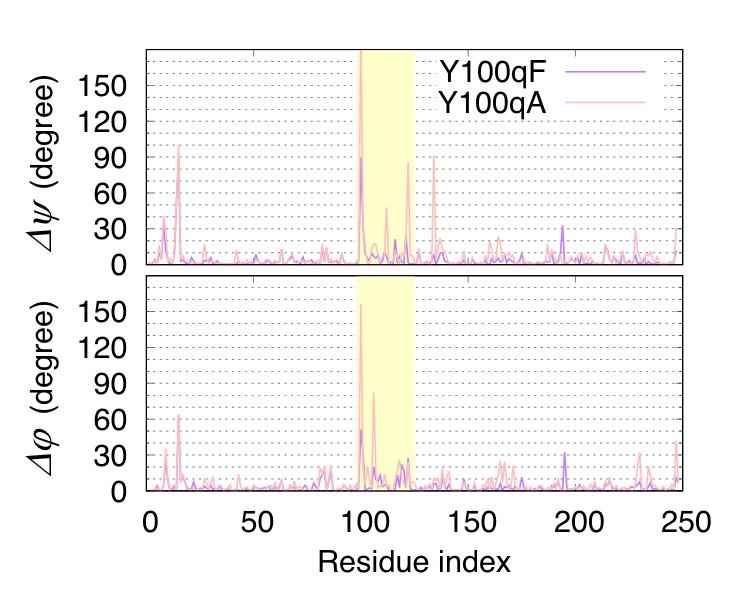


**Figure S2.** Distributions of differences in dihedral angles, Δ*ψ* (top panel) and Δ*ϕ* (bottom panel), of the whole protein for Y100qF and Y100qA are shown in pink and purple, respectively. The region shown in yellow represents the CDR-H3 residues.


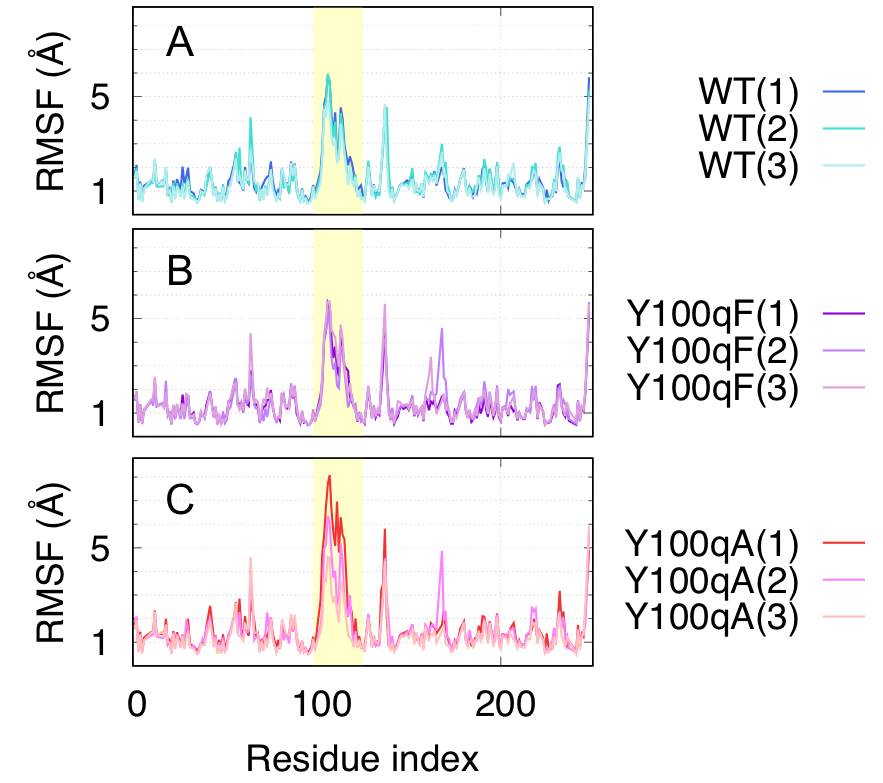


**Figure S3.** RMSF values of side-chain heavy atoms per residue for the (A) WT, (B) Y100qF, and (C) Y100qA systems. The region shown in yellow corresponds to the CDR-H3 residues.


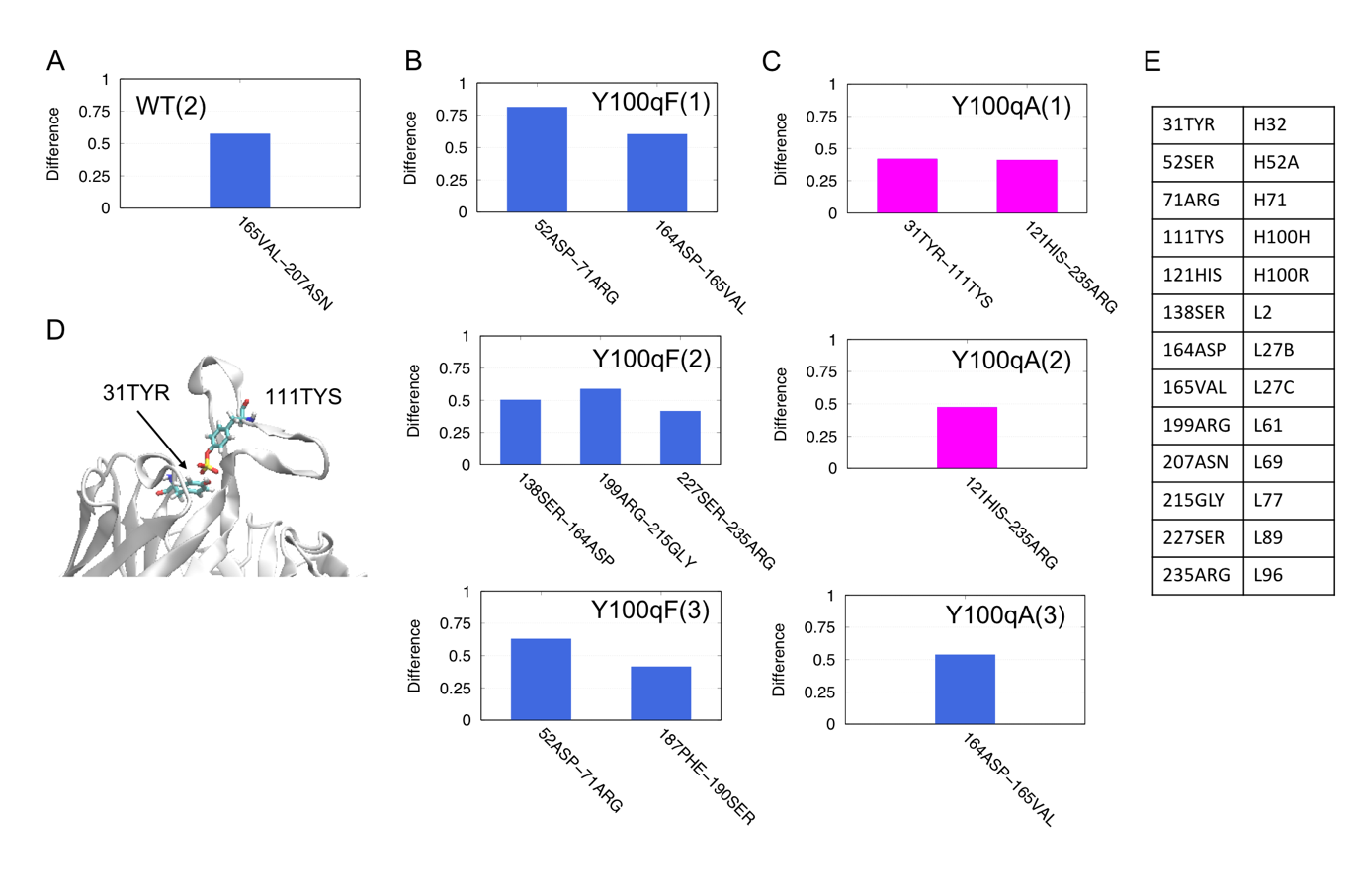


**Figure S4.** The differences in the ratios of hydrogen bond formation of (A) WT(1), (B) Y100qF(1-3), and (C) Y100qA(1-3) from the WT(3). Only the “increased pairs” (difference in ratio > 0.4) are plotted. The pairs including one or more residues in the CDR-H3 are colored in magenta. (D) The locations of 31TYR and 111TYS are represented as stick models. (E) Correspondence table of the residue indices used in A-C (left) and the Kabat numbering (right).
